# Traditional Chinese Medicine: An Exogenous Regulator of Crosstalk between the Gut Microbial Ecosystem and CKD

**DOI:** 10.1155/2022/7940684

**Published:** 2022-12-10

**Authors:** Xian Sun, Wei Sun, Yiting Huang, Jie Chen

**Affiliations:** ^1^College of Traditional Chinese Medicine, College of Integrated Chinese and Western Medicine, Nanjing University of Chinese Medicine, Nanjing 210023, China; ^2^Department of Nephrology, Jiangsu Province Hospital of Chinese Medicine, Affiliated Hospital of Nanjing University of Chinese Medicine, Nanjing 210029, China; ^3^Jiangsu Province Hospital of Chinese Medicine, Affiliated Hospital of Nanjing University of Chinese Medicine, Nanjing 210029, China; ^4^Nanjing University of Chinese Medicine Hanlin College, Taizhou 225300, China

## Abstract

Chronic kidney disease (CKD) is often accompanied by an imbalance in the gut microbial ecosystem. Notably, the imbalanced gut microbiota and impaired intestinal barrier are the keys to the crosstalk between the gut microbial ecosystem and CKD, which was the central point of previous studies. Traditional Chinese medicine (TCM) has shown considerable efficacy in the treatment of CKD. However, the therapeutic mechanisms have not been fully elucidated. In this review, we explored therapeutic mechanisms by which TCM improved CKD via the gut microbial ecosystem. In particular, we focused on the restored gut microbiota (i.e., short-chain fatty acid- and uremic toxin-producing bacteria), improved gut-derived metabolites (i.e., short-chain fatty acid, indoxyl sulfate, p-Cresyl sulfate, and trimethylamine-N-oxide), and intestinal barrier (i.e., permeability and microbial translocation) as therapeutic mechanisms. The results found that the metabolic pattern of gut microbiota and the intestinal barrier were improved through TCM treatment. Moreover, the microbiota-transfer study confirmed that part of the protective effect of TCM was dependent on gut microbiota, especially SCFA-producing bacteria. In conclusion, TCM may be an important exogenous regulator of crosstalk between the gut microbial ecosystem and CKD, which was partly attributable to the mediation of microbiota-targeted intervention.

## 1. Introduction

Chronic kidney disease (CKD) is defined as the presence of progressive and irreversible destruction of renal structure or function. It is an important public health concern, affecting 10.6%–13.4% of the general population worldwide [1]. However, risk factors for abnormal renal structure or function are diverse, and the pathogenesis has not been fully elucidated [2]. Therefore, currently available strategies for slowing the progression of CKD are few and incomplete. Clearly, additional therapeutic avenues to accessing effective treatment of CKD must be recognized, and public health strategies must be developed to overcome current barriers, including the management, control, and delay of CKD [3].

**Figure 1:**
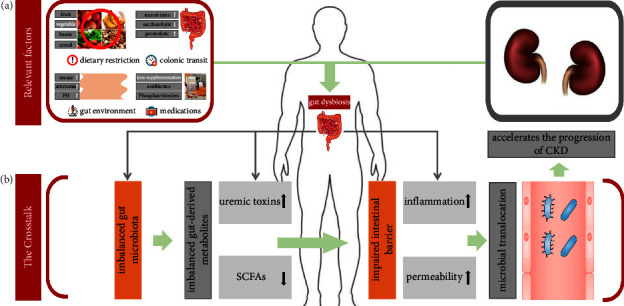
The gut microbial ecosystem and CKD. (a) Factors associated with gut dysbiosis in CKD. (b) The crosstalk between the gut microbial ecosystem and CKD.

Notably, researches in recent years have linked alterations in the gut microbiota (a condition known as “dysbiosis”) and its mediation on the intestinal barrier with chronic diseases outside the digestive system (e.g., CKD) [4, 5]. The imbalanced gut microbiota and impaired intestinal barrier are key to the crosstalk between the gut microbial ecosystem and CKD, which was the central point of previous studies (Figure 1). Briefly, CKD-related changes in gut microbiota lead to abrupt shifts in the production of gut-derived metabolites, accompanied by an impaired intestinal barrier. The alteration of the intestinal barrier allows the translocation of bacterial components from the gut into the bloodstream, ultimately contributing to renal inflammation [6, 7]. Therefore, restoring the gut microbial ecosystem (i.e., microbiota, gut-derived metabolites, and intestinal barrier) or engaging in microbiota-targeted interventions may be potential strategies for the prevention and management of CKD.

Accumulating evidence suggests that traditional Chinese medicine (TCM) has perfect therapeutic effects for alleviating diseases (e.g., diabetes, obesity, ulcerative colitis) based on gut microbiota and its metabolites [8–10]. The gut microbiota can alter the chemical composition of individual herbs or herbal extracts to have different bioavailability, bioactivity, or toxicity than their precursors. Bidirectionally, TCM herbs or herbal extracts can also remodel the diversity of gut microbiota to alleviate related diseases [11]. Several recent studies have determined that TCM can significantly influence the progression of CKD [12, 13]. However, there is still a lack of a comprehensive summary of the effects and mechanisms of TCM on CKD from the perspective of microecology. Therefore, this review focused on the core crosstalk between the gut microbial ecosystem and CKD, that is, gut microbiota and intestinal barrier, to explore the therapeutic mechanisms of TCM on CKD progression.

## 2. The Crosstalk between the Gut Microbial Ecosystem and CKD

### 2.1. The Gut Microbiota and CKD

#### 2.1.1. The Imbalanced Gut Microbiota and CKD

Symbiosis is considered a close and long-term biological interaction between two symbionts (e.g., gut microbiota and the human body). Healthy gut microbiota can produce corresponding dynamic changes with the body's biological rhythms to maintain host homeostasis. On the contrary, gut dysbiosis (e.g., altered microbiota composition and its metabolic capacity) may contribute to the development and progression of chronic diseases, including CKD. For example, changes in microbiota composition can transform normally symbiotic gut microbiota into a pathogenic factor that adversely affects renal function. Encouragingly, in recent years, large-scale clinical studies on the gut microbiota (e.g., composition, abundance, symbiotic relationship, functional prediction) of CKD patients have gradually increased [14, 15]. At the same time, breakthroughs have also been made in exploring the potential pathogenesis of CKD through animal models based on gut microbiota [16, 17]. The goal of these studies is to seek therapeutic targets that may be used to improve morbidity and survival in patients with CKD.

There is increasing evidence of gut microbiota dysbiosis in CKD. Vaziri et al. found that patients with stage V of CKD had 190 significantly different microbial taxonomic units (OTUs) compared to healthy controls. Similar results were obtained in animal experiments, that is, the model group of 5/6 nephrectomy-induced CKD rats had significant differences in bacterial OTUs compared with the sham-operated group [18].

Moreover, research on the marker microbiota and the metabolic pattern of the gut microbiota in CKD are also increasing. As reported in previous studies on animals and patients with CKD, the relative abundance of Lactobacillus was significantly reduced. In contrast, Enterobacteriaceae is overgrown with a marked increase in relative abundance[19, 20]. Jiang et al. found that the relative abundance of short-chain fatty acid (SCFA)-producing bacteria in CKD patients was significantly reduced, which promoted the metabolic pattern of the gut microbiota from saccharolytic fermentation to protein fermentation. Ultimately, these changes may cause a shift in the enterotype of CKD patients [21].

On the other hand, the bidirectionality of imbalanced gut microbiota and CKD has also been experimentally confirmed. In a study of 30 patients without receiving dialysis, bacterial DNA was detected in the blood of 6 of them (20%), and its bacterial genera were found to overgrow in the guts of these patients. In addition, these 6 patients had significantly elevated C-reactive protein and IL-6, a marker of low-grade inflammation, compared with the 24 patients in which bacterial DNA was not detected. These findings confirmed gut microbiota dysbiosis in CKD patients. Furthermore, overgrown bacteria could translocate through the gut into the bloodstream to induce low-grade inflammation and ultimately promote CKD progression [22].

Gut microbiota dysbiosis in CKD patients is closely associated with diet restrictions, medications, slow colonic transit, and changes in the gut environment (Figure 1(a)). The above four points are not only attached to the background of CKD but also the trigger factors of imbalanced gut microbiota in CKD patients. Specifically: (1) Dietary restriction: dietary fiber generally refers to the nondigestible carbohydrates present in food. Foods rich in dietary fiber include fruits, vegetables, beans, whole grains, etc. High dietary fiber intake can reduce the substrate required for protein fermentation, and reduce colonic transit time by stimulating intestinal mucosa to increase secretion and promote intestinal motility [23]. For the general population, the current recommended dietary fiber intake is 20–30 g/d [24]. For CKD patients, there are no specific recommended doses in related guidelines. High dietary fiber intake will increase potassium and phosphorus levels, leading to imbalanced electrolytes in CKD patients. Therefore, these patients are generally characterized by reduced dietary fiber intake. However, insufficient intake can induce the imbalance of saccharolytic and proteolytic microbiota, leading to a shift in the metabolic pattern from saccharolytic fermentation to protein fermentation [25]. Ultimately, two major gut-derived metabolites, SCFAs and gut-derived uremic toxins (GDUT) are deregulated [26]. (2) Medications: CKD patients are often exposed to antibiotics to treat vascular access infections or other infectious diseases. However, antibiotics can deplete key bacterial taxa that maintain gut homeostasis, while reducing bacterial diversity and metabolic capacity [27]. On the other hand, for CKD patients with anemia or calcium-phosphorus metabolism disorders, the long-term administration of iron supplementation or phosphate binders may induce changes in the gut environment and affect the colonization of microbiota, leading to imbalanced gut microbiota [28, 29]. (3) Slow colonic transit: prolonged colonic transit time can reduce the availability of carbohydrates in the colon, thereby inducing an increase in proteolytic microbiota, and ultimately leading to the imbalance of saccharolytic and proteolytic microbiota in CKD patients [30]. (4) Changes in the gut environment: urea concentrations are significantly elevated in CKD patients [31]. It has been confirmed that the increased influx of urea into the intestinal lumen contributes to the proliferation of urease-producing bacteria [32, 33]. Lau et al. confirmed that the relative abundance of urease-producing bacteria was significantly increased in CKD patients (stage V) compared with healthy controls [34]. Urea is decomposed by urease to produce ammonia. Ammonia raises the pH of the intestinal lumen and alters the composition of the gut microbiota, leading to gut dysbiosis [35].

#### 2.1.2. The Imbalanced Gut-Derived Metabolites and CKD

**Figure 2:**
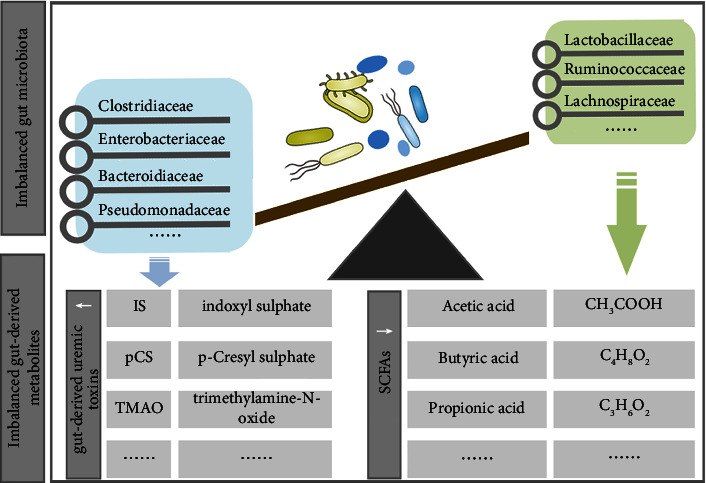
Imbalanced gut microbiota and gut-derived metabolites in CKD.

CKD-related gut microbiota dysbiosis favored the overgrowth of GDUT-producing bacteria with proteolytic activity, while significantly inhibiting the expansion of beneficial bacteria with saccharolytic activity (e.g., SCFA-producing bacteria) [36]. As a result, the most representative gut-derived metabolites, namely, GDUT and SCFAs, were dysregulated (Figure 2).

According to the source of uremic toxins, it can be divided into three categories: (1) Endogenous metabolites (urea and creatinine, etc.). (2) Exogenous ingested substances (oxalate, etc.). (3) Gut-derived metabolites, namely, GDUT, including indoxyl sulfate (IS), p-Cresyl sulfate (pCS), and trimethylamine-N-oxide (TMAO) [37]. As for SCFAs, they are considered to be the end products of bacterial fermentation, which mainly include acetate, propionate, and butyrate [38].

**Figure 3:**
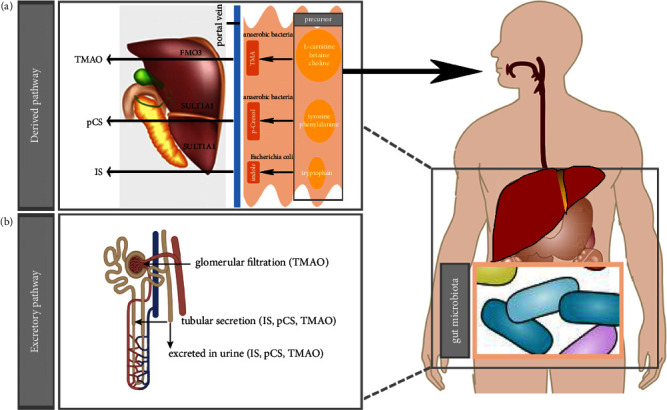
Schematic diagram of the derivation and excretion of gut-derived uremic toxins (GDUT). (a) The derivation pathway of GDUT. (b) The excretory pathway of GDUT. IS, indoxyl sulfate; pCS, p-Cresyl sulfate; TMAO, trimethylamine-N-oxide.

IS and pCS: specifically, dietary tryptophan is catabolized into indole by gut *Escherichia coli* under the action of tryptophanase. After indole is absorbed from the gut into the portal circulation, it is converted to hydroxyindole and IS by two hepatic cytochrome oxidases, CYP 2E1 and SULT1A1, respectively. As for pCS, dietary tyrosine and phenylalanine are catabolized by gut anaerobic bacteria to 4-hydroxyphenylacetic acid, and then decarboxylated to p-cresol, which is converted to pCS by SULT1A1 in the liver [39]. For details, see Figure 3(a). Serum IS and pCS concentrations were observed to be extremely low in healthy populations, around 10 *μ*mol and 60 *μ*mol, respectively. Both are mainly excreted by renal tubular secretion (Figure 3(b)) under normal renal function [40]. However, IS and pCS cannot be effectively eliminated in the state of renal dysfunction, resulting in a large accumulation. In ESRD patients, the concentrations of both could be 10–50 times higher than those in healthy controls [41]. The key toxic effects of IS and pCS on renal cells mainly include induction of oxidative stress [42], increased inflammatory response [43], enhanced profibrotic expression [44], and downregulated expression of nephroprotective proteins (e.g., Klotho protein) [45]. IS and pCS are protein-bound uremic toxins that bind tightly through albumin-binding site II with up to 90% binding. The current clinical dialysis strategies are extremely limited in the clearance of these two uremic toxins [46, 47].

TMAO: The main sources of TMAO are L-carnitine, choline, and betaine. These precursors are metabolized by gut microbiota to trimethylamine (TMA). The absorbed TMA enters the liver through the portal venous circulation and is rapidly oxidized to TMAO by flavin monooxidase (FMO3) [48]. See Figure 3(a) for details. TMAO is associated with an increased risk of cardiovascular disease and the progression of CKD. Notably, cardiovascular disease is the leading cause of death in CKD patients [49]. TMAO is normally excreted by glomerular filtration and tubular secretion (the main pathway) (Figure 3(b)), and then excreted in the urine [50]. Circulating TMAO concentrations gradually increased with the progression of CKD. A previous study found that patients with CKD (stages III-V) had higher plasma TMAO concentrations than non-CKD subjects [15]. Compared with patients with CKD (stage IIIb), patients with CKD (stage IV) had higher plasma TMAO concentrations [51]. The serum TMAO concentration of ESRD patients was 20 times higher than that of healthy controls [52]. The serum TMAO concentration of patients who successfully received renal transplantation could quickly return to the normal range [53]. Notably, unlike IS and pCS, TMAO can be effectively removed by conventional dialysis [54].

Notably, although the small intestine provides the main site for the host's digestive activities, the production site of SCFAs is mainly concentrated in the colon, especially the ascending colon [55]. SCFA-producing bacteria, such as Lactobacillaceae, Ruminococcaceae, and Lachnospiraceae, can effectively degrade nondigestible carbohydrates to produce SCFAs [56]. Most of them can be rapidly absorbed by the intestinal epithelium through specific transporters or by diffusion, and are the energy source suppliers of colon tissue [57]. Among them, acetate is an important cofactor for bacterial growth [58]. Propionate and butyrate are key metabolites that provide the primary energy source for the colonocytes [59]. Most of the absorbed SCFAs are used as energy sources [60], while a small part is consumed by the liver [61]. Ultimately, the remaining SCFAs can pass through the circulatory system to target organs and tissues, where they can perform certain functions [62]. Mechanistic studies continue to provide evidence for the importance of SCFAs in diseases (e.g., hypertension, inflammatory bowel disease, and CKD) [63–65]. Therefore, the homeostasis of SCFAs may provide clues and evidence for the balance between the gut microbiota and the host. At present, an increasing number of studies have focused on the interplay among SCFAs, intestinal barrier, and CKD [66, 67]. Significantly decreased SCFA concentrations were observed in CKD patients compared to healthy controls [68]. Recent evidence suggested that concentrations of SCFAs, especially acetate and butyrate, are almost completely suppressed in patients and animal models with CKD [69, 70]. In addition, there is increasing evidence that reduced concentrations of SCFAs contribute to renal dysfunction [71]. Conversely, supplementation with SCFAs, especially butyrate, can improve the intestinal barrier and control microbial translocation, and ultimately achieve nephroprotective effects [72]. Therefore, targeting the gut microbiota, especially SCFA-producing bacteria, may provide a promising therapeutic approach for CKD. The mechanism by which SCFAs improve the intestinal barrier will be elaborated in section 3.1 of this review.

### 2.2. The Impaired Intestinal Barrier and CKD

**Figure 4:**
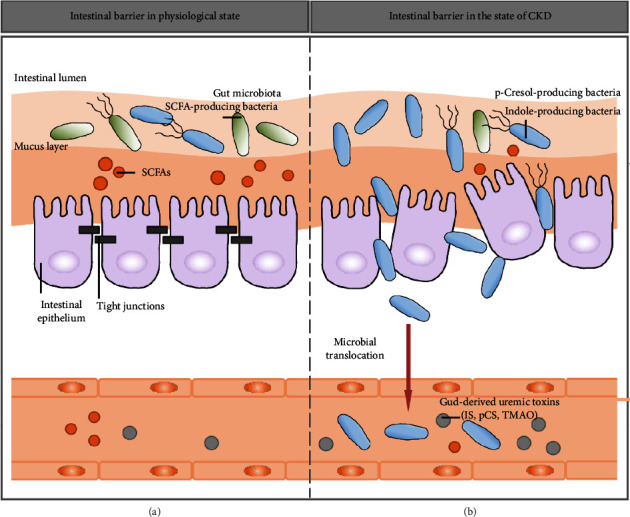
Impaired intestinal barrier and CKD [7].

The intestinal epithelium is a single layer of columnar epithelium that separates the intestinal lumen from the lamina propria. It plays an important role in nutrient absorption while acting as a natural barrier to prevent and inhibit microbial translocation. These columnar epithelial cells are adjacent to each other by tight junctions, forming the “seal” of the intestinal barrier [73]. In a healthy population, the characteristics of gut ecosystem homeostasis include the following: (1) The gut microbiota structure is characterized by the predominance of commensal bacteria (e.g., SCFA-producing bacteria, etc.), accompanied by few pathogenic bacteria (e.g., p-Cresol- and indole-producing bacteria). (2) The intestinal barrier structure and function are intact (Figure 4(a)).

The intestinal barrier of CKD was shown in Figure 4(b). Due to factors such as diet restrictions, medications, slow colonic transit, and changes in the gut environment, drastic changes in the gut microbiota of CKD patients are caused. Imbalanced gut microbiota can further lead to an impaired intestinal barrier (characterized by increased intestinal permeability) and microbial translocation. Ultimately, the translocated bacterial components can flow into the kidney through systemic circulation, exacerbating renal inflammation. The specific mechanisms of impaired intestinal barrier caused by the imbalanced gut microbiota in CKD are as follows: (1) CKD patients have significantly elevated urea, which diffuses into the intestinal lumen and further contributes to the expansion of urease-producing bacteria. Urea is hydrolyzed by urease to produce ammonia, which results in increased ammonia production in the intestinal lumen due to unregulated urease. This results in increased intestinal PH and a damaged intestinal wall, ultimately leading to increased intestinal permeability [33]. (2) Imbalanced SCFA-producing bacteria and reduced concentration of SCFAs resulted in a dramatic reduction in the nutrient and energy sources of colon tissue. Theoretically, these changes could lead to an impaired intestinal barrier [74]. (3) Impaired intestinal barrier stimulates leukocyte infiltration. Local inflammation and associated proinflammatory cytokines induced the endocytosis of intestinal epithelial tight junction proteins, which further contributes to increased intestinal barrier permeability [75].

## 3. Mechanisms of TCM in the Treatment of CKD via the Gut Microbial Ecosystem

### 3.1. The Potential Therapeutic Mechanisms

TCM treatment could improve the clinical symptoms and renal function indexes of CKD patients. Previous animal experiments also found that CKD progression could be delayed by TCM treatment, which was characterized by improved renal function (pathological) indicators and systemic inflammation. In addition, the regulatory effects of TCM on the gut microbial ecosystem had also been confirmed (Table 1). Notably, the above studies provided evidence that the protective effect of TCM was partially attributable to the mediation of the gut microbial ecosystem. Therefore, TCM may be an important exogenous regulator of crosstalk between the gut microbial ecosystems and CKD.

**Figure 5:**
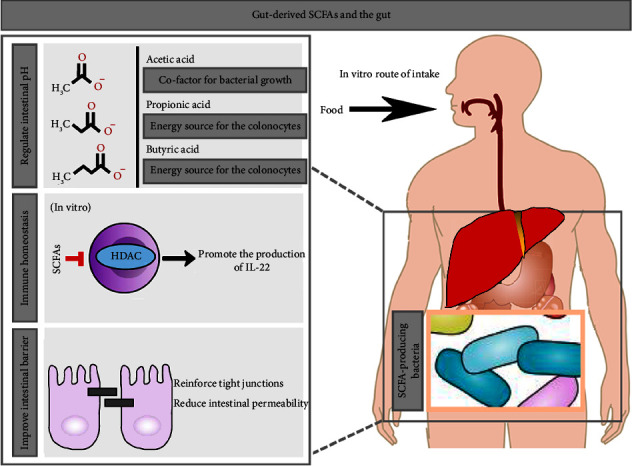
Effects of gut-derived SCFAs on the gut.

The mechanisms of TCM in the treatment of CKD via the gut microbial ecosystem were reviewed as follows:

1. *Improvement of Imbalanced Gut Microbiota*. Jianpi Yishen Decoction (JPYS) is composed of 8 single TCM herbs, namely, *Astragali radix* (Huangqi, HQ), Atractylodis Macrocephalae Rhizoma (Baizhu, BZ), Dioscoreae Rhizoma (Shanyao, SY), Cistanches Herba (Roucongrong, RCR), Amomi Fructus Rotundus (Doukou, DK), Salviae Miltiorrhizae Radix et Rhizoma (Danshen, DS), Radix Rhei Et Rhizome (Dahuang, DH), and Glycyrrhizae Radix et Rhizoma Praeparata cum Melle (Zhigancao, ZGC). A recent study showed that JPYS had significant effects on improving renal function and modulating gut microbiota in CKD rats. Specifically, JPYS increased the relative abundance of SCFA-producing bacteria (Coprococcus, Phascolarctobacterium, and Parasutterella), whereas the relative abundance of GDUT-producing bacteria (*Clostridium* XIVb) was decreased. The metabolic pattern of gut microbiota shifted from saccharolytic fermentation to protein fermentation, which contributed to the imbalanced SCFA and GDUT-producing bacteria in CKD [76]. Therefore, improving the imbalance between SCFA- and GDUT-producing bacteria may play a role in the treatment of CKD.
2. *Regulation of Imbalanced Gut-Derived Metabolites*. Ji et al. preliminarily confirmed that significantly elevated TMAO levels were observed in 5/6 nephrectomized rats, and rhubarb enema could effectively reduce circulating TMAO and alleviate renal function in CKD rats, which may be related to the regulation of TMAO-producing bacteria (*Intestinimonas*, *Methanobrevibacter, Parasutterella, Anaerostipes, Catabacter, Ruminiclostridium*, *Desulfovibrio,* and *Clostridia*) [17]. TMAO, IS, and pCS are the most representative gut-derived uremic toxins in CKD. GDUTs could directly act on renal cells by inducing oxidative stress, increasing inflammatory response, enhancing profibrotic expression, and downregulating the expression of nephroprotective protein levels. Notably, the current clinical dialysis strategies are extremely limited in the clearance of IS and pCS.
3. However, Based on the gut microbial ecosystem, previous studies have continuously provided clues and scientific evidence that improved gut microbiota and intestinal barrier may be important entry points for CKD treatment. Preliminary studies had found that SCFAs, as important metabolites of gut microbiota, participate in the aforementioned processes, and the specific manifestations were as follows: (1) Imbalanced SCFA-producing bacteria in CKD: the metabolic pattern of gut microbiota shifted from saccharolytic fermentation to protein fermentation, which contributed to the inhibition of SCFAs. (2) The gut is affected by SCFAs (Figure 5). (3) Regulate intestinal pH value: provide a suitable environment for the production of acetate, propionate, and butyrate, which is conducive to shaping a perfect gut microbial ecosystem. For example, butyrate is the main energy substrate of colonocytes, providing about 70% of the important energy required for cell growth and differentiation. Propionate is also an energy source for colonocytes, which has the effects of regulating cholesterol levels and antilipogenesis. Furthermore, acetate acts as the predominant SCFA, which is an important cofactor for bacterial growth [80]. (3) Maintain intestinal immune homeostasis: IL-22 produced by innate lymphocytes (ILCs) and CD4 T cells is critical for intestinal immunity. Yang et al. found that gut-derived SCFAs could activate GPR41 and inhibit histone deacetylase, thereby promoting the production of IL-22 by CD4 T cells and ILCs to maintain intestinal immune homeostasis and alleviate colitis in mice [81]. (4) Improve the intestinal barrier: SCFAs can activate *G* protein-coupled receptors (GPCRs), inhibit histone deacetylases, and increase the expression levels of the intestinal tight junction. This in turn reduces intestinal permeability. A recent study found that 12 weeks of *Lycium ruthenicum* anthocyanins supplementation in high-fat diet-induced mice could induce the production of SCFAs by regulating the gut microbiota, thereby attenuating intestinal barrier dysfunction [82]. The relationship between SCFAs and the gut is not limited to this, and related clinical and animal studies have been advancing in recent years. (5) Concentration of SCFAs affects CKD progression. Reduced concentration of SCFAs led to renal dysfunction. Conversely, supplementation with SCFAs, especially butyrate, could improve the intestinal barrier and control microbial translocation, and ultimately achieve nephroprotective effects [83]. Hence, targeting the gut microbiota, especially SCFA-producing bacteria, may provide a new strategy for the treatment of CKD.
4. Improvement of the impaired intestinal barrier. The imbalanced gut microbiota could further lead to an impaired intestinal barrier (characterized by increased intestinal permeability) and microbial translocation. The specific mechanisms by which the imbalanced gut microbiota in CKD leads to impaired intestinal barrier mainly include elevated urea, decreased SCFA concentrations, and local inflammation of the intestinal wall. Ultimately, the translocated bacterial components can flow into the kidney through systemic circulation, exacerbating renal inflammation. A recent study confirmed that rhubarb enema could reduce renal interstitial fibrosis and delay the progression of CKD. Specifically, rhubarb increased the SCFA-producing bacteria (*Akkermansia muciniphila*, *Lactobacillus acidophilus*, *Bacteroides caccae*, and *Faecalibaculum rodentium*) in CKD rats, thereby increasing SCFA (propionic acid, butyric acid) concentrations and ultimately contributing to an improved intestinal barrier and controlled gut microbiota [67].

### 3.2. Representative TCM : Fuzheng Huayu Jiangzhu Tongluo Prescription and Yishen Qingli Heluo Granule

Represented by Fuzheng Huayu Jiangzhu Tongluo prescription (FZHY) and Yishen Qingli Heluo granule (YQHG), the mechanisms of TCM in the treatment of CKD via the gut microbial ecosystem were elaborated.

**Figure 6:**
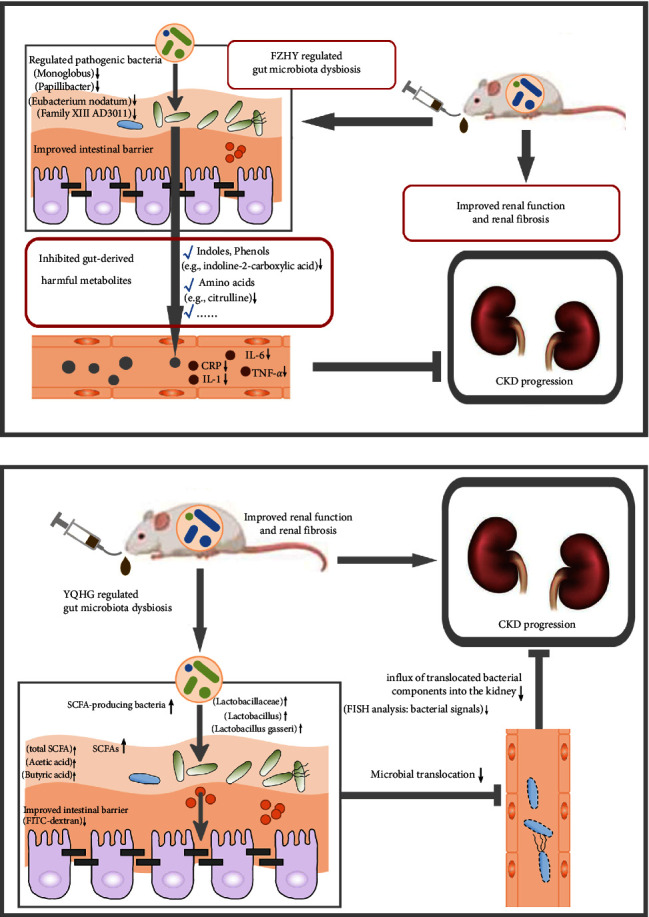
Representative TCM : Fuzheng Huayu Jiangzhu Tongluo prescription (FZHY) and Yishen Qingli Heluo granule (YQHG). (a) Schematic diagram of therapeutic mechanisms of FZHY to alleviate CKD via gut microbiota and its related metabolites [78]. (b) Schematic diagram of therapeutic mechanisms of YQHG to alleviate CKD via gut microbiota and intestinal barrier [79].

FZHY is composed of 9 single TCM herbs, namely, Radix Rhei Et Rhizome (Dahuang, DH), *Astragali radix* (Huangqi, HQ), *Radix rehmanniae praeparata* (Shudihuang, SDH), *Slauia miltiorrhiza Bunge* (Danshen, DS), *Carthamus tinctorius* L. (Honghua, HH), *Hirudo* (Shuizhi, SZ), Eupolyphaga (Tubiechong, TBC), *Scutellariae radix* (Huangqin, HQin), and *Glycyrrhizae radix et rhizoma* (Gancao, GC), which has been used in clinical practice for a long time. An Animal experiment found [78] that FZHY treatment hindered disease progression in CKD rats, manifested as improvements in renal function and fibrosis, decreased expression of renal fibrosis-related indicators (LN, FN, Col-I, Col-III), and systemic inflammation markers (CRP, TNF-*α*, IL-6, IL-1). In addition, FZHY significantly reduced the pathogenic bacteria (Monoglobus, Papillibacter, *Eubacterium nodatum*, Family XIII AD3011) and the precursor of gut-derived uremic toxins, and upregulated the expression of intestinal tight junction proteins (ZO-1, Occludin, Claudin-1). Elevated ammonia levels had been shown to promote disruption of the intestinal barrier. A previous study found that Monoglobus was positively correlated with blood ammonia levels. The inhibition of Monoglobus by FZHY may have a protective effect on the intestinal barrier, which was consistent with the increased expression of intestinal tight junction proteins in this study. In addition, increased GDUT-related bacteria (Family XIII AD3011) or metabolites (indoles, phenols, etc.) act on renal cells and contribute to renal fibrosis and inflammation, ultimately promoting CKD progression. In short, the underlying mechanism of FZHY alleviating CKD is mainly through the interrelationship between gut microbiota and gut-derived metabolites (Figure 6(a)).

**Figure 7:**
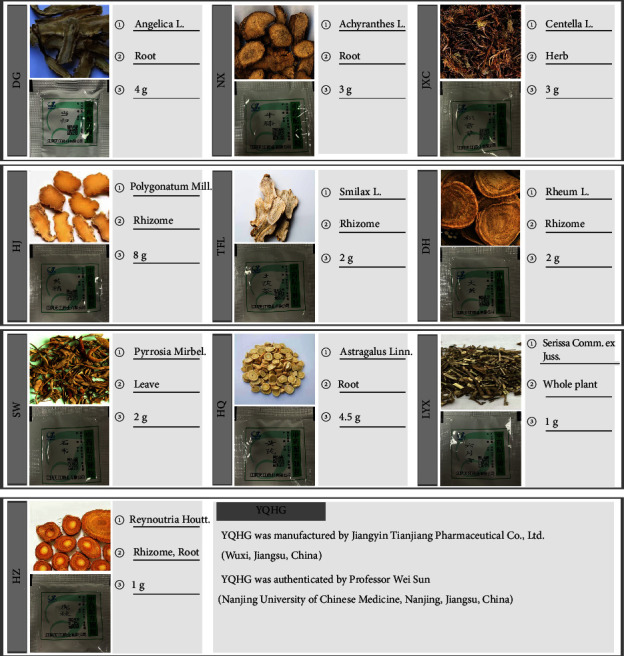
Details of Yishen Qingli Heluo Granule (YQHG) [12]: (1) the genus; (2) parts used; (3) dose used (g).

YQHG was composed of 10 single TCM granules, namely, *Angelicae sinensis radix* (Danggui, DG), *Achyranthis bidentatae radix* (Niuxi, NX), *Centella asiatica* (L.) Urban (Jixuecao, JXC), *Polygonati rhizoma* (Huangjing, HJ), *Smilacis glabrae rhixoma* (Tufuling, TFL), *Radix rhei et rhizome* (Dahuang, DH), *Pyrrosiae folium* (Shiwei, SW), *Astragali radix* (Huangqi, HQ), *Serissa japonica* (Thunb.) Thunb (Liuyuexue, LYX), and *Polygoni cuspidati rhizoma et radix* (Huzhang, HZ). All the granules were authenticated by Professor Wei Sun (Nanjing University of Chinese Medicine, Nanjing, Jiangsu, China). For details, see Figure 7.

Clinical studies had shown that clinical symptoms and Scr levels in CKD patients could be improved by YQHG. In addition, YQHG also delayed progression from stage III to stage IV in CKD patients [84].Sun et al. showed [79] that YQHG treatment significantly halted the progression of CKD, characterized by increased body weight, improved renal appearance and function, and reduced tissue damage in 5/6 nephrectomized rats. Importantly, the study demonstrated that 5/6 nephrectomized rats treated with YQHG showed significant improvement in renal fibrosis, such as reduced glomerular and tubulointerstitial fibrosis areas. Notably, they found that YQHG modulated bacterial communities, particularly increasing the relative abundance of SCFA-producing bacteria (i.e., Lactobacillaceae, Lactobacillus, and *Lactobacillus gasseri*), which in turn improved SCFA (i.e., total SCFA, acetic acid, butyric acid) concentrations and intestinal barrier (decreased FITC-dextran concentration). Ultimately, controlled microbial translocation (reduced bacterial signals) contributes to alleviating renal inflammation (reduction of IL-6 expression) (Figure 6(b)). Interestingly, to further confirm the importance of the gut microbiota for YQHG in CKD treatment, they reshaped the bacterial community by conducting a microbiota-transfer study (cohousing and fecal microbiota transplantation). Impressively, the kidneys of CKD rats were profoundly protected after the microbiota-transfer study, characterized by the remission of renal inflammation, fibrosis, and dysfunction. The results suggested that the protective effect of YQHG was partly attributable to the mediation of gut microbiota, especially SCFA-producing bacteria.

## 4. Conclusions

In this review, we explored the therapeutic mechanisms of TCM to improve CKD via the gut microbial ecosystem. We summarized from the following three aspects: (1) TCM could regulate the metabolic pattern of gut microbiota: the metabolic pattern of gut microbiota shifted from saccharolytic fermentation to protein fermentation through TCM treatment. Specifically, TCM treatment contributed to elevated SCFA and reduced GDUT. (2) TCM could improve the intestinal barrier: TCM increased SCFA concentrations (i.e., total SCFA, acetic acid, butyric acid), which in turn improved the intestinal barrier. Ultimately, controlled microbial translocation contributed to alleviating renal inflammation. (3) Therapeutic effect mediated by the gut microbiota: the microbiota-transfer study confirmed that the protective effects of TCM were partly attributable to the mediation of gut microbiota, especially SCFA-producing bacteria (i.e., Lactobacillaceae, Lactobacillus, and *Lactobacillus gasseri*). These findings propose a microbiota-targeted intervention and suggest that TCM may be a promising therapeutic avenue for overcoming current CKD-related barriers.

## Figures and Tables

**Table 1:** Changes in the gut microbial ecosystem and therapeutic effects.

TCM	Treatment	Subjects	Effects on the gut microbiota	Effects on the gut-derived metabolites	Effects on the intestinal barrier	Therapeutic effects	References
Jianpi Yishen decoction	Oral gavage (10.89 mg/kg, once a day for 12 weeks)	Male Sprague–Dawley rats	(1) Regulated the SCFA-producing bacteria (Coprococcus↑, Phascolarctobacterium↑, Parasutterella↑); (2) regulated the uremic-toxin-producing bacteria (clostridium XIVb↓)	N/A	N/A	(1) Improved renal function (BUN↓, UALB↓); (2) restored levels of CKD complication-related indicators (blood reticulocyte↓, serum calcium↑)	Zhang et al. [76]
Rhubarb granules	Enema (2.12 g/kg, once a day for 4 weeks)	Male Sprague–Dawley rats	(1) Modified the diversity of gut microbiota (Shannon index↓); (2) regulated the relative abundance of gut microbiota (*Clostridium↑, Alistipes↑, Sutterella*↑, S24-7 from *Bacteroidales*↑, one member from Clostridiaceae↑, and one member from Enterobacteriaceae↑)	N/A	Improved intestinal barrier integrity: (1) H&E staining of colon tissue (the edema in the lamina propria and mucosal layer↓, the infiltration of inflammatory cells in the mucosal layer↓); (2) the expression of key indicators of the intestinal barrier integrity (Occludin↑, Claudin-1↑, ZO-1↑); (3) the expression of TLR4 signaling pathways in the colon tissue (TLR4↓, NF-*κ*B↓, pNF-*κ*B↓, MyD88↓)	(1) Improved renal function (Scr↓); (2) improved renal fibrosis; (3) Improved systemic inflammation (IL-1*β*↓, IL-6↓, LPS↓)	Ji et al. [77]
Rhubarb granules	Enema (1) low-dose (1.05 g/kg, once a day for 8 weeks) and (2) high-dose (2.10 g/kg, once a day for 8 weeks)	Male Sprague–Dawley rats	Regulated the TMAO-related gut microbiota (*Intestinimonas↓, Methanobrevibacter↓, Parasutterella↓, Anaerostipes↓, Catabacter↓, Ruminiclostridium↓, Desulfovibrio↓, and Clostridia*↓)	TMAO↓	N/A	(1) Improved renal function (Scr↓, serum urea↓); (2) H&E staining of renal tissue (renal tubule atrophy↓, monocyte infiltration↓, renal interstitial fibrosis↓); (3) immunohistochemistry staining of renal tissue (FN↓, *α*-SMA↓, COl-I↓); (4) improved systemic inflammation (IL-6↓, TNF-*α*↓, IFN-*γ*↓)	Ji et al. [17]
Rhubarb granules	Enema (0.2 g/ml, once a day for 4 weeks)	Male Sprague–Dawley rats	Regulated the SCFA-producing bacteria (*Akkermansia muciniphila↑, Lactobacillus acidophilus*↑, *Bacteroides caccae*↑, and *Faecalibaculum rodentium*↑)	SCFAs↑	Improved intestinal barrier integrity: (1) H&E staining of colon tissue (intestinal mucosal inflammation and edema↓, the height of intestinal mucosal villi↑); (2) electron microscope of colon tissue (microvilli, tight junctions, desmosomes, and mitochondrial structure were improved); (3) regulate the expression of key indicators of the intestinal barrier integrity (ZO-1↑, Occludin↑)	(1) Improved renal function (Scr↓, BUN↓, urine protein creatinine ratio↓); (2) improved systemic inflammation (IL-1*β*↓, TNF-*α*↓, IFN-*γ*↓); (3) H&E staining of renal tissue (different degrees of renal tubule brush margin shedding and atrophy↓, mononuclear lymphocytes infiltration in tubulointerstitium↓, tubulointerstitial fibrosis)	Ji et al. [67]
Yishen Qingli Heluo granule	Oral gavage (1) low-dose (1.4 g/kg, once a day for 8 weeks); (2) middle-dose (2.8 g/kg, once a day for 8 weeks); (3) high-dose (5.6 g/kg, once a day for 8 weeks)	Male Sprague–Dawley rats	(1) regulated the relative abundance of gut microbiota (Firmicutes↓, Bacteroidota↑); (2) the ratio of F/B↓	N/A	N/A	(1) Increased the body weight of rats; (2) improved kidney appearance (color, capsule, border); (3) improved renal function (Scr↓, BUN↓, 24h urinary protein↓); (4) histopathologic evaluation of renal tissue a) H&E: inflammation infiltration↓, mesangial expansion↓, tubular atrophy and dilation↓, glomerular sclerosis↓, interstitial fibrosis↓) and b) histopathological indicators: (Glomerular fibrosis area↓, tubulointerstitial fibrosis area↓); (5) regulates markers of inflammation and fibrosis in renal tissue (PTGS2↓, IL-6↓)	Sun et al.[12]
Fuzheng Huayu Jiangzhu Tongluo Fang	Oral gavage (4.92 g/kg, once a day for 7, 14, and 21 days, respectively)	Male Sprague–Dawley rats	(1) Modified the diversity of gut microbiota; (2) regulated the relative abundance of pathogenic bacteria/uremic-toxin-producing bacteria (Monoglobus↓, Papillibacter↓, *Eubacterium nodatum*↓, Family_XIII_AD3011↓)	Regutlated the precursor of gut-derived uremic toxins (4-(3,4-dihydro-2h-1,5-benzodioxepin-7-yl)-2-methyl-1,3-thiazole↓, indoline-2-carboxylic acid↓),	Improved intestinal barrier integrity: (1) histopathologic evaluation of colon tissues 1) H&E (epithelial cell damage↓, goblet cell reduction↓); 2) Masson: colon fibrosis↓); (2) regulate the expression of key indicators of the intestinal barrier integrity (ZO-1↑, Occludin↑, Claudin-1↑); (3) immunohistochemistry staining of colon tissue (ZO-1↑, Occludin↑, Claudin-1↑)	(1) Improved renal function (Scr↓, BUN↓); (2) histopathologic evaluation of renal tissue a) H&E (structure damage↓, renal tubule dilatation↓, partial renal tubule epithelial cell shedding↓, hyperemia↓) and b) Masson (renal fibrosis↓); (3) regulated the expression of the renal fibrosis-related markers (LN↓, FN↓, Col-I↓, Col-III↓); (4) immunohistochemistry staining of renal tissue (LN↓, FN↓, Col-I↓, Col-III↓); (5) improved systemic inflammation (CRP↓, TNF-*α*↓, IL-6↓, IL-1↓)	Chen et al. [78]
Yishen Qingli Heluo granule	Oral gavage (5.6 g/kg, once a day for 8 weeks)	Male Sprague–Dawley rats	Regulated the relative abundance of SCFA-producing bacteria (Lactobacillaceae↑, Lactobacillus↑, lactobacillus gasseri↑)	Regulated the SCFA concentrations (total SCFA↑, acetic acid↑, butyric acid↑)	(1) Improved intestinal permeability (FITC-dextran↓); (2) improved microbial translocation (FISH analysis: bacterial signals↓)	(1) Increased the body weight of rats; (2) improved kidney appearance (color, capsule, border); (3) improved renal function (Scr↓, BUN↓, 24 h urinary protein↓); (4) histopathologic evaluation of renal tissue; 1) H&E (inflammation infiltration↓, mesangial expansion↓, tubular atrophy and dilation↓, glomerular sclerosis↓, and interstitial fibrosis↓); 2) histopathological indicators (glomerular fibrosis area↓, tubulointerstitial fibrosis area↓); (5) regulates markers of inflammation in renal tissue (IL-6↓); (6) microbiota-transfer study showed that the protective effect of Yishen Qingli Heluo granule was partly attributed to the mediation of the gut microbiota, especially the SCFA-producing bacteria	Sun et al. [79]

## Abbreviations

SCFA: Short-chain fatty acid
TMAO: Trimethylamine-N-oxide
F/B: Firmicutes/bacteroidota
ZO-1: Zonula occludens-1
FISH: Fluorescence in situ hybridization
BUN: Blood urea nitrogen
UALB: Urinary albumin
Scr: Serum creatinine
IL-1*β*: Interleukin-1beta
IL-6: Interleukin-6
IL-1: Interleukin-1
LPS: Lipopolysaccharide
LN: Laminin
FN: Fibronectin
*α*-SMA: Alpha-smooth muscle actin
Col-I: Collagen-I
Col-III: Collagen-III
TNF-*α*: Tumor necrosis factor-alpha
IFN-*γ*: Interferon gamma
PTGS2: Prostaglandin-endoperoxide synthase 2
CRP: C-reactive protein.
